# Molecular Mechanisms of Synaptic Plasticity: Dynamic Changes in Neuron Functions

**DOI:** 10.3390/ijms241612567

**Published:** 2023-08-08

**Authors:** Giuseppina Martella

**Affiliations:** 1 Laboratory of Neurophysiology and Plasticity, IRCCS Fondazione Santa Lucia, 00143 Rome, Italy; g.martella@hsantalucia.it; 2 Department of Human Sciences, Faculty of Umanities Educations and Sports, Pegaso University, 80143 Naples, Italy

The human brain has hundreds of billions of neurons and at least 7 million dendrites have been hypothesized to exist for each neuron, with over 100 trillion neuron–neuron, neuron–muscle, and neuron–endocrine cell synapses [1,2].

Our body continually receives stimuli from the outer environment, and our brain’s ability to respond to these stimuli is ensured through synaptic processes, motivating the foundations of this Special Issue.

This issue aims to underline the role of synaptic plasticity phenomena in our body, and clarify the mechanism operated by neurons to guarantee these phenomena. The collection of the issue comprises 14 papers, including 8 reviews and 6 original works, of which is a protocol for differentiating neurons from human stem cells and 5 are preclinical works.

Of these preclinical manuscripts, one is focalized on the glucocorticoids that may alter the gene and protein expression in catecholamine neurons. Using organotypic cultures incubated with the neurosteroid corticosterone, the authors of the paper demonstrated significant increases in tyrosine hydroxylase and dopamine transporter; conversely, modifications were recorded in phenylethanolamine N-methyltransferase. This demonstrated that dopamine signals may carry out regulations via internal glucocorticoid secretion [3].

In a mouse model of Parkinson’s disease, Imbriani and coworkers showed that caspase-3, considered an effector caspase involved in neuronal apoptosis, has a hormesis-based double role in PINK1-knockout (KO) mice. In other words, when this caspase has a lower level of activation, it modulates physiological phenomena, as well as corticostriatal LTD, and only at higher activation levels is it conducive to the apoptotic process. In PINK1-KO mice, lower caspase-3 activation is capable of rescuing a defective LTD by promoting dopamine release [4].

Professor Petrosini’s group demonstrates that the pathway of the hippocampus, dorsal striatum, and amygdala shows a high expression of receptors for cannabinoid type 1 (CB1). The presence of these receptors in areas deputed to spatial learning depicts the involvement of cannabinoid in navigational strategies. The authors demonstrated that treatment with the CB1 antagonist, AM251, impaired spatial learning and modified the pattern of performed navigational strategies in a mouse model of Gaucher disease. The strength of the synaptic plasticity on the behavioral output is modulated by CB1 receptors, which leads to spatial navigational strategies [5].

Dystonia is a movement disorder characterized by the co-contraction of agonist and antagonistic muscles that cause body twisting and related pain. The pathophysiology of the disease was partly related to the downward regulation and dysfunction of the dopamine D2 receptor in the striatum. Using a mouse model of DYT1 dystonia, D’Angelo and collaborators found that DYT1 mutant mice present a reduced expression of D2 receptors associated with a marked reduction in the number and size of D2-positive synapses at the level of striatal area. This rearrangement causes a longer duration and greater sphere of influence of dopamine transmission, explaining the electrophysiological characteristics of this pathology [6].

SNAP-25 is a component of the SNARE complex of proteins that have the function of coupling the synaptic vesicle and plasma membranes together, in order to favor the neurotransmitter release in the nervous system. Moreover, SNAP-25 influences synaptic plasticity in the first stages of life. 

In mice of 30 days, transgenic for an unmatured isoform of SNAP-25b, Schaffer collateral CA1 synapses had a much faster kinetic release with a consequent decrement in LTP and an enhancement in LTD amplitude. Conversely, mice four-month-old mice showed that the learning process avoiding LTD upregulation rescued physiological plasticity. These data explain the correlation between long-term synaptic plasticity and cognitive learning ability [7].

Starting from two neural stem cell lines from healthy patients, Capetian et al. have identified a cultural technique that integrates and contrasts the best neuronal elements in their protocol paper. Differentiated neuronal stem cells were incorporated into a highly concentrated Matrigel (neurosphere) droplet. After inclusion, neurospheres were treated with a combination of glutaraldehyde and paraformaldehyde using various membrane combinations to optimize imaging via transmission electron microscopy. This new method represents the best way to analyze synaptic structures [8].

The reviews in this Special Issue aim to highlight the synaptic capacities of our brain in relation to their molecular mechanisms, emphasizing structural and functional changes due to plasticity.

Inhibitory networks involved in synaptic plasticity were shown to be regulated by a vast number of pathways in their synaptic connections. Because inhibitory plasticity was characterized later than excitatory synaptic plasticity, this literature revision proposed by Mapelli represents a new and interesting point of view to understand synaptic mechanisms. The authors proposed an overview of inhibitory plasticity in different brain areas [9].

Through an in-depth study of synapses, it was found that the neurocentric model, encompassing the neuronal pre- and post-synaptic terminals and the synaptic cleft, is not able to explain all the fine-tuned plastic modifications visualized in pathological and physiological events. For this reason, the tripartite synapse model including oligodendrocytes, astrocytes, and microglia was proposed. Moreover, physiological synaptic plasticity and maladaptive plasticity commonly showed a deep connection with other molecular elements of the extracellular matrix.

Alongside that, a new model of the tetrapartite synapse, including the neurovascular unit and the immune system, was recently proposed. The latter appears more congruent with the different mechanisms of physiologic adaptive and maladaptive plasticity. However, a better interpretation may be granted by the construction of predictive molecular models [10].

N-methyl-d-aspartate glutamate receptors (NMDARs) are tetramers composed of different homologous subunits of GluN1-, GluN2-, or GluN3-type, able to generate a high variety of receptor subtypes with various pharmacological and signaling properties. Their subunit composition is regulated during the development of activity-dependent synaptic plasticity. Through their literature revision, Franchini and coworkers showed the role of these subunits in both physiology and pathological synaptic plasticity [11].

Recently, 5-HT7R serotonin receptors have been shown to be involved in reshaping neuronal cytoarchitecture in many neurodevelopmental disorders. Over the past few years, the synaptic plasticity of this receptor has been demonstrated in the ILD and LTP forms. Furthermore, 5-HT7R contributes to inflammatory bowel disease. Perrone-Capano’s group offers a review in which the new aspects highlighted in the digestive tract and immune system have been clarified [12].

Neuregulins (NRGs), a family of proteins acting on tyrosine kinase receptors of the ErbB family play an essential role in the development of the nervous system. They also contribute to the functioning of the adult brain through synaptic plasticity. Ledonne and Mercuri presented a literature review that emphasizes the role of GRN signaling in modulating synaptic plasticity. Furthermore, they suggest that NRG-dependent dysregulation of synaptic plasticity could be implicated in numerous pathophysiological diseases [13].

Anti-homeostatic synaptic plasticity, first LTP, represents one of the main physiological mechanisms used by our brain in response to brain damage. Both anti-homeostatic and homeostatic synaptic mechanisms contribute to shaping brain networks. In multiple sclerosis, inflammatory mediators induce altered synaptic functioning that corresponds to the addition of demyelination and gray matter atrophy. In the revision proposed by Stampanoni Bassi and colleagues, an altered LTP expression was shown to contribute to disrupting the brain network [14].

Different forms of synaptic plasticity have been established: anti-homeostatic (i.e., Hebbian) and homeostatic plasticity (i.e., synaptic scaling). The balance between these forms is necessary to correspond to the architecture of the brain system.

In the review made by Stampanoni Bassi and co-workers, the key features of the brain network architecture were introduced, which resulted from fine-tuning between two different forms of synaptic plasticity [15].

Inflammatory/immunity mediators are closely related to diseases of the central nervous system. Moreover, these mediators are involved in synaptic plasticity pathways and synaptic plasticity is hardly affected by the immune system. Additionally, the immune system is linked to synaptic processes.

The two main custom clearance systems are autophagy and ubiquitin–protease (UPS). In their review, Fornai et al. discuss the role of autophagy and UPS in connecting immunity with synaptic plasticity in health and disease [16].

At present, the increase in neurological and neuropsychiatric diseases urges understanding the function of the brain network and the fine mechanism underlying brain dysfunction, which this Special Issue aims to satisfy.
